# C2 pedicle screw placement on 3D-printed models for the performance assessment of CTA-based screw preclusion

**DOI:** 10.1186/s13018-023-03498-x

**Published:** 2023-01-03

**Authors:** Yuelin Wu, Zhaoquan Liang, Junhao Bao, Ling Wen, Li Zhang

**Affiliations:** 1 The Second School of Clinical Medicine, Southern Medical University, Guangzhou, Guangdong Province China; 2 The Spine Department, Orthopaedic Center, Guangdong Second Provincial General Hospital, Guangzhou, Guangdong Province China

**Keywords:** C2 pediculoisthmic component, 3-D printing, C2 pedicle screw, Multiplanar reconstruction, High-riding vertebral artery

## Abstract

**Background:**

3-D printing technology has a large spectrum of applications in upper cervical spinal surgery, but none have evaluated the radiological analysis of the feasibility of C2 pedicle screw placement. Thus, this study aimed to perform 3.5-mm-diameter C2 pedicle screw placement on models for performance assessment of CTA-based preoperative screw preclusion.

**Methods:**

We enrolled 152 patients who underwent CTA of the cervical spine between April 2020 and December 2020. Transverse pediculoisthmic width (TPW), oblique pediculoisthmic width (OPW), minimum pediculoisthmic diameter (MPD), internal height, and isthmus height were measured preoperatively. Subsequently, 1:1 3D-printed bone models were created, and a 3.5-mm-diameter C2 pedicle screw was placed on the models. All 3D-printed models underwent postoperative CT multiplanar reconstruction to evaluate the screw trajectory for the performance assessment of CTA-based preoperative screw preclusion.

**Results:**

The ROC curves of the MPD, TPW, OPW, Internal height and Isthmus height showed that the optimal cutoff values for each of the five groups were measured values of 4.78, 4.44, 4.37, 4.22 and 5.59 mm, respectively. The AUC, sensitivity, and specificity of MPD were 0.992, 95.1% and 100%, respectively. The MPD had higher metrics than the TPW (AUC, 0.949; sensitivity, 87.9%), internal height (AUC, 0.885; sensitivity, 80.8%; specificity, 84.6%), and isthmus height (AUC, 0.941; sensitivity, 87.2%). We found no evidence of a difference between MPD and OPW in terms of the AUC and sensitivity (0.93 and 95.5%, respectively).

**Conclusions:**

C2 pedicle screw placement on 3D-printed models is useful for performance assessment of CTA-based preoperative screw preclusion. MPD measurement with CTA multiplanar reconstruction showed the best performance for judging acceptable or unacceptable screws. However, the definition of HRVA could be modified by a 4.2 mm-internal height or by measuring only the isthmus height for judging the preclusion of C2 pedicle screw placement.

## Background

The C2 pedicle screw is the preferred technique for posterior atlantoaxial internal fixation because of its good biomechanical properties and high fusion rate [1–3]. The placement of C2 pedicle screws carries the risk of iatrogenic vertebral artery injury, which can lead to breach of the vertebral artery groove, insufficient blood supply to the basilar artery, and even death [4]. Measurements in axial CT images, oblique axial CT images, and the definition of high-riding artery (HRVA) are commonly used to evaluate the feasibility of C2 pedicle screw placement to avoid complications of vertebral artery injury [5–7]. However, the accuracy of conventional CT methods has not been verified, and it is unknown whether the definition of HRVA is still applicable to judge the preclusion of C2 pedicle screw placement. Multiplanar reconstruction (MPR) on computed tomography angiography (CTA) can be used to simulate the C2 pedicle screw trajectory, and help surgeons observe the narrowest section of the C2 pediculoisthmic component (PIC), which is considered an accurate method for the preoperative evaluation of the feasibility of safe C2 pedicle screw placement [8–11]. However, the evaluation performance of CTA multiplanar reconstruction remains unclear.

Recently, 3D printing technology has a large spectrum of applications in upper cervical spinal surgery including the creation of patient-specific drill guide templates and 3D-printed vertebral body implants [12–17]. Furthermore, surgeons can visualize the screw entry point, trajectory, length, pedicle diameter, thickness, and anatomical deformities using 3D-printed cervical models. In this study, measurements of axial CTA images, oblique axial CTA images, MPR-CTA images, and definition of HRVA were performed to preoperatively evaluate the feasibility of C2 pedicle screws, 3D-printed bone models of C2 vertebrae were created and C2 pedicle screws were placed on the models for performance assessment of CTA-based preoperative screw preclusion.

## Materials and methods

### Patients

One-hundred-fifty-two patients who underwent CTA examination of the cervical spine at our hospital between April 2020 and December 2020 were enrolled, including 87 male (57.2%) and 65 female (42.8%), with an average age of 59.36 ± 13.73 years (range 16–87 years) at the time of CT scanning.

### Preoperative measurements

**Fig. 1:**
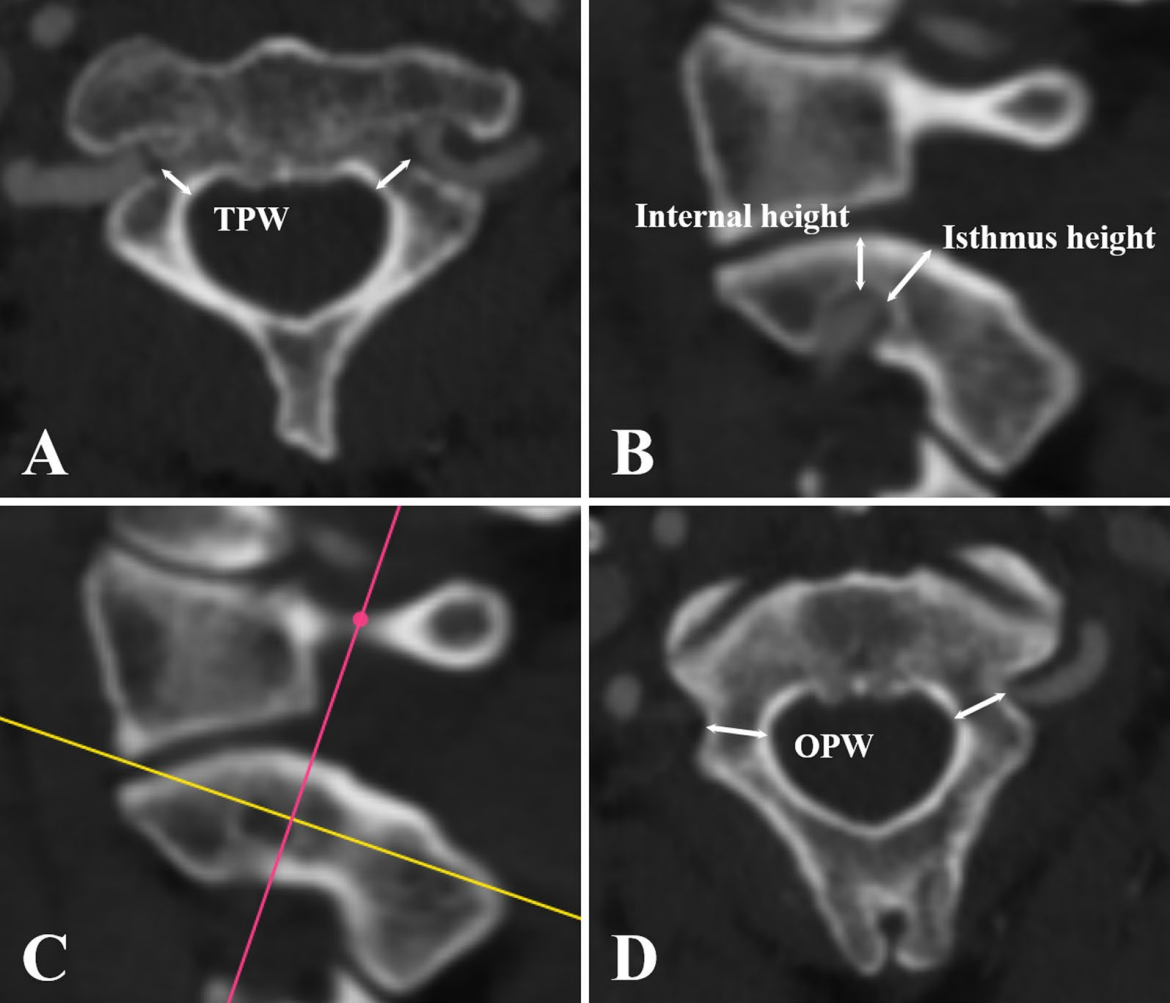
Measurement of TPW (**A**), internal height, isthmus height (**B**) and OPW (**C** and **D**)

**Fig. 2:**
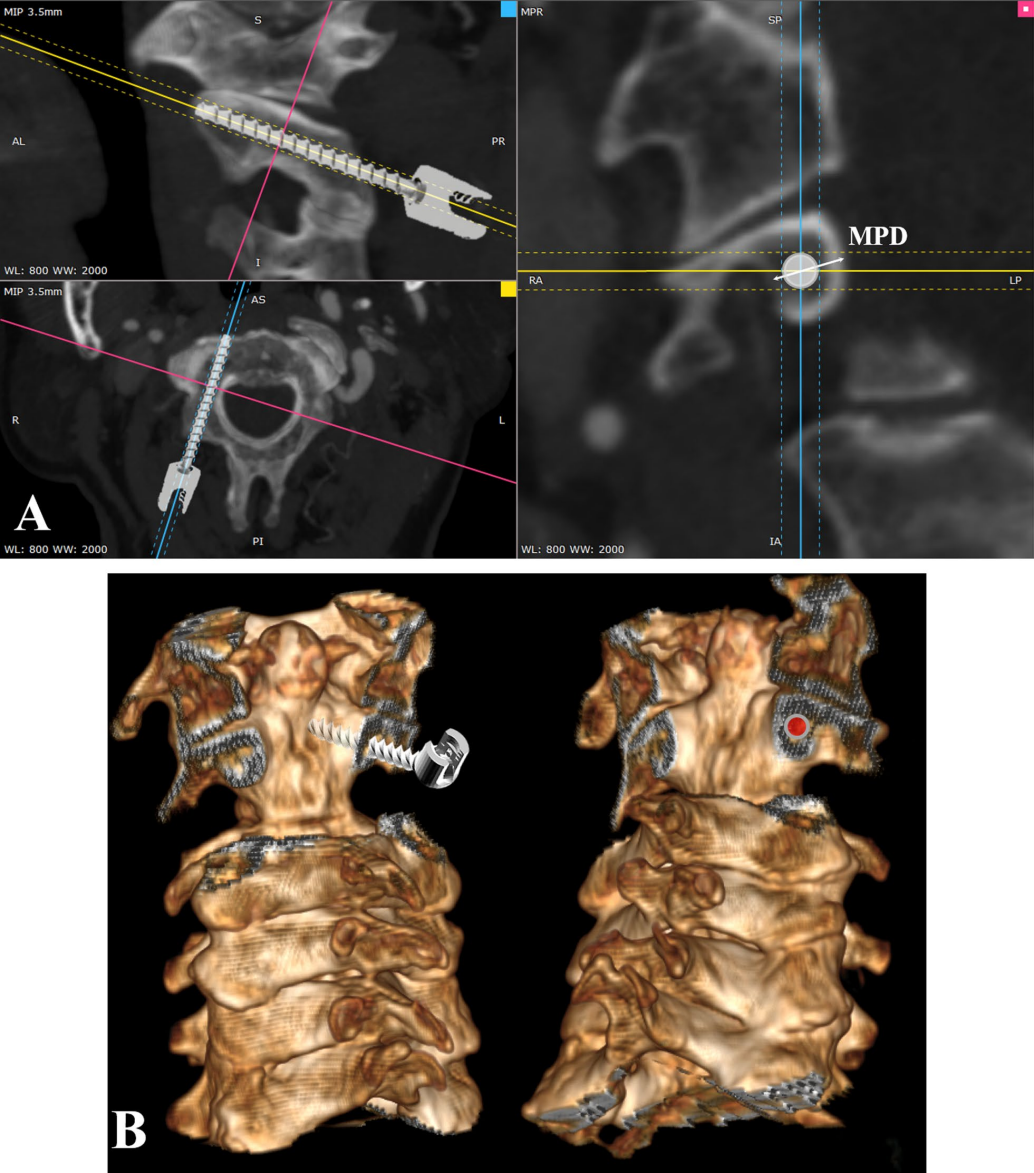
(**A)** Preoperative CTA multiplanar reconstruction was performed to measure MPD and evaluate the feasibility of C2 pedicle screw placement. (**B**) The measurement plane was shown on 3D reconstruction, which is perpendicular to the screw trajectory of both the axial and sagittal planes

The DICOM data were entered into RadiAnt DICOM Viewer software (Medixant, Poznan, Poland). Preoperative evaluation was performed as follows: transverse PIC width (TPW) was measured on the orthogonal axial plane (Fig. 1A); internal height and isthmus height were measured on an orthogonal sagittal image 3 mm lateral to the cortical margin of the spinal canal wall at C2 (Fig. 1B). The oblique PIC width (OPW) was measured on oblique axial CTA images parallel to the sagittal pedicle axis using multiplanar reconstruction (Fig. 1C and D). A 3.5-mm-diameter C2 pedicle screw placement was simulated using multiplanar reconstruction combined with maximum intensity projection (MIP). The minimum PIC diameter (MPD) was measured at the narrowest sections of the PIC perpendicular to the pedicle axis in both the axial and sagittal planes (Fig. 2).

### Operation procedure on the 3D-printed models and screw placement evaluation

**Fig. 3:**
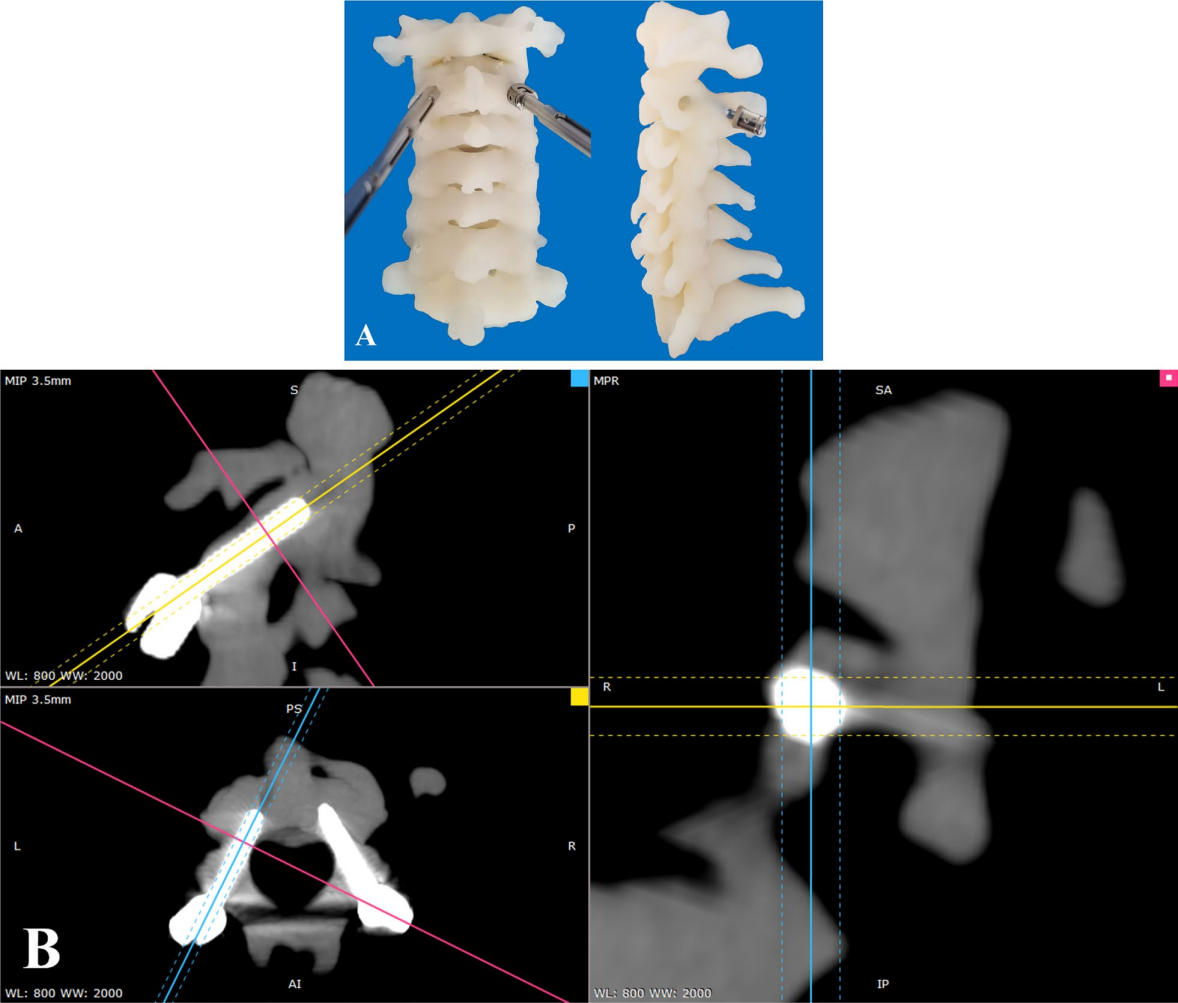
(**A**) C2 pedicle screws were inserted into 3D-printed bone model. (**B**) Postoperative CT multiplanar reconstruction was performed to assess the screw trajectory and cortical breach

DICOM data were imported into the Mimics software (version 23.0; Materialize, Belgium) to reconstruct and generate a 3D bone model of the C2 vertebrae in the STL format. 1:1 physical bone models were created using a 3D printer (Stratasys J850, Stratasys Inc., Eden Prairie, MN, USA) and 3D printing materials (BoneMatrix RGD516, Stratasys Inc., Eden Prairie, MN, USA). The BoneMatrix materials can accurately mimic bone density characteristics, biomechanical characteristics and behave like native bone when force is applied such as drilling, reaming, or sawing. A 3-mm-diameter electric drill was used to drill slowly along the axis of the pedicle. A spherical probe was used to determine the integrity of the screw trajectory. A screw tap was then used to create a screw hole, and a 3.5-mm-diameter C2 pedicle screw was placed in the hole, according to the screw trajectory planned preoperatively (Fig. 3A). All 3D-printed models underwent postoperative CT MPR to evaluate the feasibility of 3.5-mm-diameter C2 pedicle screw placement, and to determine whether the screw entered the C2 vertebrae through the pediculoisthmic component (Fig. 3B). Then, according to whether the screw penetrated the cortical bone, C2 pedicle screw placement was evaluated using the grading method proposed by Sciubba et al. [18]: grade 0: the screw was completely wrapped in the cortical bone without penetrating the cortex; grade I: the percentage of screw diameter beyond the cortical edge was less than 25%; grade II: the percentage was between 26 and 50%; grade III: the percentage was between 51 and 75%; and grade IV, the percentage was between 76 and 100%. When the evaluations were inconsistent, group consensus was reached through discussion.

### Statistical analysis

Statistical analyses were performed using SPSS Statistics for Windows (version 26.0, IBM, NY, USA) and MedCalc for Windows (version 19.0.1; MedCalc Software). The areas under the receiver operating characteristic curves (AUCs) with 95% CIs were compared using the DeLong test for the MPD, TPW, OPW, internal height, and isthmus height. The performance metrics (sensitivity, specificity, positive predictive value, and negative predictive value) of MPD, TPW, OPW, internal height, and isthmus height were evaluated and compared using McNemar’s test. The optimal cutoff value for each CTA method was set at the highest Youden index to discriminate between acceptable and unacceptable screws. *P*-values less than 0.05 were considered to indicate statistical significance.

## Results

### Preoperative measurements and cortical breach in 3D-printed bone models

The data for the radiographic measurements and breached screws in the models are summarized in Table 1. The mean transverse PIC width (5.48 ± 1.49 mm) was measured narrower than the minimum PIC diameter (6.54 ± 1.91 mm, *P* < 0.001). The mean isthmus height (5.48 ± 1.49 mm) was greater than both minimum PIC diameter (6.54 ± 1.91 mm, *P* < 0.001) and internal height (6.50 ± 3.13 mm, *P* < 0.001). No significant differences were found between the minimum PIC diameter, oblique PIC width, and internal height. A significant difference was noted between the left and right MPD, OPW, and isthmus height measurements (*P* < 0.05).

**Table 1:** Summary of the preoperative measurements and breached screws

Characteristic	Value
*Preoperative measurements*	
MPD in millimeter	6.54 ± 1.91
L	6.74 ± 1.73
R	6.34 ± 2.06*
TPW in millimeter	5.48 ± 1.49†
L	5.69 ± 1.44
R	5.27 ± 1.52
OPW in millimeter	5.56 ± 1.67
L	5.96 ± 1.65
R	5.15 ± 1.60*
Internal height in millimeter	6.50 ± 3.13
L	6.47 ± 2.96
R	6.53 ± 3.30
Isthmus height in millimeter	7.55 ± 2.78†
L	7.76 ± 2.56
R	7.34 ± 2.98*
Cortical breach (% of total screws)	39 (12.83%)
Left (% of breached screws)	12 (30.77%)
Right (% of breached screws)	27 (69.23%)*
*Breach grade*	
I	22 (56.42%)
II	9 (23.08%)
III	5 (12.81%)
IV	3 (7.69%)

†Data are for comparison with MPD (*P* < 0.05)

*Data are for comparison with left side (*P* < 0.05)

One hundred and fifty-two models underwent 3.5-mm-diameter C2 pedicle screw placement, with 39 total breaches (12.83%) and 27 breaches (69.23%) on the right side, which was higher than the 12 breaches (30.77%) on the left side (*P* < 0.05). The magnitude of the breach was classified as I in 22 cases (56.42%), II in nine cases (23.08%), III in five cases (12.81%), and IV in three cases (7.69%).

### Performance of MPD, TPW, OPW, Internal height and Isthmus height for evaluating acceptable and unacceptable screws

**Fig. 4:**
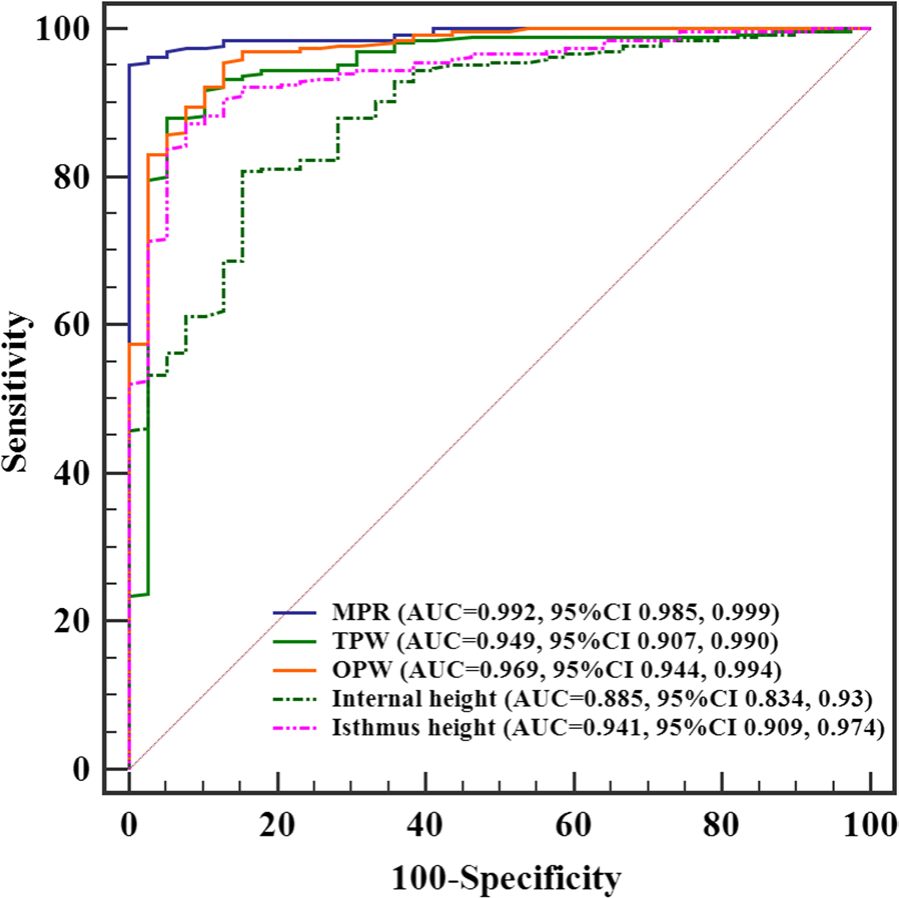
ROC curve of the MPD, TPW, OPW, Internal height and Isthmus height

The receiver operating characteristic curves of the MPD, TPW, OPW, Internal height and Isthmus height showed that the optimal cutoff values for each of the five groups were measured values of 4.78, 4.44, 4.37, 4.22 and 5.59 mm, respectively. If the measurements were greater than the cutoff values, the feasibility of C2 pedicle screw placement was evaluated as acceptable; otherwise, it was considered unacceptable. The AUC, sensitivity, specificity, positive predictive value, and negative predictive value of MPD were 0.992 (95% CI: 0.985, 0.999), 95.1% (252 of 265 screws), 100% (39 of 39 screws), 100% (252 of 252 screws), and 75% (39 of 52 screws), respectively. MPD had higher metrics than TPW (AUC, 0.949 [*P* = 0.037]; sensitivity, 87.9% [233 of 265 screws; *P* < 0.001]), internal height (AUC, 0.885 [*P* < 0.001]; sensitivity, 80.8% [214 of 265 screws; *P* < 0.001]; specificity, 84.6% [33 of 39 screws; *P* < 0.001]), and isthmus height (AUC, 0.941 [*P* = 0.0013]; sensitivity, 87.2% [233 of 265 screws; *P* < 0.001]). The specificity of MPD was significantly higher than that of OPW (84.6% [34 of 39 screws; *P* < 0.001]), although we found no evidence of a difference in the AUC and sensitivity between MPD and OPW (0.93 [*P* = 0.06] and 95.5% [253 of 265 screws; *P* > 0.1], respectively) (Table 2, Fig. 4).

**Table 2:** Performance of MPD, TPW, OPW, Internal height and Isthmus height for evaluating acceptable and unacceptable screws

Parameter	MPD	TPW	OPW	Internal height	Isthmus height
AUC	0.992	0.949†	0.969	0.885†	0.941†
95% CI	0.985, 0.999	0.907, 0.990	0.944, 0.994	0.834, 0.936	0.909, 0.974
Sensitivity (%)	95.1 (252/265)	87.9 (233/265)†	95.5 (253/265)	80.8 (214/265)†	87.2 (231/265)†
Specificity (%)	100.0 (39/39)	94.9 (37/39)	87.2 (34/39)†	84.6 (33/39)†	92.3 (36/39)
PPV (%)	100.0 (252/252)	99.1 (233/235)	98.1 (253/258)	97.3 (214/220)	98.7 (231/234)
NPV (%)	75.0 (39/52)	53.6 (37/69)	73.9 (34/46)	39.3 (33/84)	51.4 (36/70)
Youden index J	0.951	0.828	0.826	0.885	0.79
Criterion (mm)	4.78	4.44	4.37	4.22	5.59

Except were indicated, numbers in parentheses are numbers of screws. *AUC*  area under the receiver operating characteristic curve, *NPV*  negative predictive value, *PPV*  positive predictive value

†Data are for comparison with MPD (*P* < 0.05)

## Discussion

Screw fixation of the upper cervical spine requires experienced surgeons, and the preparation of cadaver spines is expensive and limited, making it difficult to obtain a large number of samples to sufficiently represent the broad spectrum of patient anatomy and pathology. Since 3D printing has become a useful tool for creating replicas for surgical simulation and surgeons can perform operations on 3D-printed models without worrying about complications, many studies focusing on surgical training and education have emerged, but none have evaluated the conventional radiological analysis of the feasibility of C2 pedicle screw placement. In this study, we preoperatively measured the parameters using the MPD, TPW, OPW, internal height and isthmus height on CTA images; a 3.5-mm-diameter C2 pedicle screw was placed on a 3D-printed bone model, and postoperative CT multiplanar reconstruction was performed to evaluate the screw trajectory and cortical breach. Given that some model materials may influence the feeling during screw insertion and the accuracy of cortical breaches due to uniform structure, the BoneMatrix materials were used in this study for creating 3D-printed bone model to replicates cortex thickness and biomechanical characteristics, which allow the screw thread to cut into part of the model cortex. The thirty-nine screws (12.83%) breached the cortex of the C2 PICs, 31 of 39 screws (79.5%) were located < 50% of the screw diameter beyond the cortical edge, and 27 of 39 screws (69.23%) had cortical breaches on the right PICs, which matched the narrower measurements of the right MPD, OPW and isthmus height.

We regarded whether there was cortical breach after screw placement as a binary classification, the performance assessment of MPD, TPW, OPW, internal height and isthmus height was done with the ROC curve. MPD achieved an AUC of 0.992 and a sensitivity of 95.1% in the judgment of acceptable and unacceptable C2 pedicle screws, which exceeded those of the TPW, internal height, and isthmus height. Previous studies have reported that preoperative measurements with multiplanar CT reconstruction can be used to accurately evaluate the suitability of C2 pedicle screw placement. Yuan et al. [8] first reported a morphological study of C2 PIC using multiplanar CT reconstruction, in which 11.7% of C2 pedicles displayed thin paries that were unsuitable for pedicle screw placement. Davidson et al. [11] reported that planning C2 pedicle screw placement with multiplane submillimeter CT reformatting allowed for a more accurate evaluation of screw fixation safety. Our study further clarified the better performance of MPD for preoperative evaluation compared with conventional CTA measurement of TPW. Moreover, the AUC of OPW and MPD showed no significant difference, and the measurement of OPW was useful by adjusting the gantry angle to match the axis of the pedicle if the conditions of multiplanar reconstruction were not available [6, 19].

Controversy continues over the criteria for defining a narrow pedicle. Maki et al. [9] showed that safe C2 pedicle screw insertion was not considered feasible in 45 (22.5%) pedicles that were ≤ 4 mm in width of the medullary cavity as measured by CT MPR. Marques et al. [10] used the MPR function of OsiriX to judge if the pedicle is feasible for screwing; they considered that for a 3.5 or 4.0 mm screw, the width of the C2 pedicle ought to arrive at a minimum of 5.5 or 6.0 mm, respectively. Our results showed that the optimal cutoff values for MPD, TPW and OPW were measured at values of 4.78, 4.44 and 4.37 mm, respectively. Therefore, C2 pedicle screw could be precluded for avoiding the risk of cortical breach when MPD was measured at less than 4.78 mm, TPW was measured at less than 4.44 mm, and OPW was measured at less than 4.37 mm.

The high-riding vertebral artery was originally defined based on the feasibility of C1-C2 transarticular screw placement [20, 21]. According to Yeom et al. [5], there is an HRVA when an internal height of 2 mm or less and/or an isthmus height of 5 mm or less on a sagittal image that is 3 mm lateral to the cortical margin of the spinal canal wall at C2. However, it is unknown whether the definition of HRVA is applicable for judging the preclusion of C2 pedicle screw placement. In the present study, the AUC, sensitivity and specificity of internal height were significantly lower than those of MPD, and the cut-off value of internal height was measured at 4.22 mm, which is much greater than the original definition of 2 mm. In addition, the isthmus height with a cut-off value of 5.59 mm arrived at an AUC of 0.941 and a specificity of 92.3%, which is equivalent to the TPW. Thus, we suggest that the definition of HRVA could be modified by a 4.2 mm-internal height or only measuring isthmus height for judging the preclusion of C2 pedicle screw placement.

In terms of limitations, screw insertion on 3D-printed models was performed by several senior surgeons, and different experiences may have affected the accuracy of the screw placement. When screw malposition results from technical problems, the models are reprinted and the screws are reinserted. Secondly, although the BoneMatrix materials are able to mimic bone density and replicate cortex thickness, the X-ray absorptivity between model cortex and model bone marrow is indistinguishable, and we considered it inessential for evaluating the screw trajectory and the screw diameter beyond the cortical edge. Thirdly, we did not consider the direction of screw insertion on CTA MPR images preoperatively and postoperatively. However, an anatomical study comparing the measurements of different CT methods was not the goal of this study. The first step of the current study was to verify the accuracy of the CTA MPR. Future research should focus on planning an ideal starting point and screw trajectory using CTA MPR, which is expected to be clinically applicable. Despite these limitations, to our knowledge, this study is the first to perform C2 pedicle screw placement on 3D-printed bone models to assess the performance of CTA-based preoperative screw preclusion.

## Conclusions

C2 pedicle screw placement on 3D-printed bone models is useful for performance assessment of CTA-based preoperative screw preclusion. MPD measurement with CTA multiplanar reconstruction showed the best performance for judging acceptable or unacceptable screws. However, the definition of HRVA could be modified by a 4.2 mm-internal height or by measuring only the isthmus height for judging the preclusion of C2 pedicle screw placement.

## Data Availability

The data and materials contributing to this article may be made available upon request by sending an e-mail to the corresponding author.
